# Biomechanical properties of native and cultured red blood cells–Interplay of shape, structure and biomechanics

**DOI:** 10.3389/fphys.2022.979298

**Published:** 2022-08-16

**Authors:** Claudia Bernecker, Maria Lima, Tatjana Kolesnik, Annika Lampl, Catalin Ciubotaru, Riccardo Leita, Dagmar Kolb, Eleonore Fröhlich, Peter Schlenke, Gerhard A. Holzapfel, Isabel Dorn, Dan Cojoc

**Affiliations:** ^1^ Department for Blood Group Serology and Transfusion Medicine, Medical University of Graz, Graz, Austria; ^2^ CNR-IOM, National Research Council of Italy - Institute of Materials, Trieste, Italy; ^3^ University of Trieste, Physics Department, Trieste, Italy; ^4^ Core Facility Imaging, Center for Medical Research, Medical University of Graz, Graz, Austria; ^5^ Core Facility Ultrastructure Analysis, Center for Medical Research, Medical University of Graz, Graz, Austria; ^6^ Gottfried Schatz Research Center for Cell Signaling, Metabolism and Aging, Division of Cell Biology, Histology and Embryology, Medical University of Graz, Graz, Austria; ^7^ Institute of Biomechanics, Graz University of Technology, Graz, Austria; ^8^ Department of Structural Engineering, Norwegian University of Science and Technology, Trondheim, Norway

**Keywords:** red blood cells, *ex vivo* erythropoiesis, biomechanical properties, optical tweezers, atomic force microscopy, digital holographic microscopy

## Abstract

Modern medicine increases the demand for safe blood products. *Ex vivo* cultured red blood cells (cRBC) are eagerly awaited as a standardized, safe source of RBC. Established culture models still lack the terminal cytoskeletal remodeling from reticulocyte to erythrocyte with changes in the biomechanical properties and interacts with membrane stiffness, viscosity of the cytoplasm and the cytoskeletal network. Comprehensive data on the biomechanical properties of cRBC are needed to take the last step towards translation into clinical use in transfusion medicine. Aim of the study was the comparative analysis of topographical and biomechanical properties of cRBC, generated from human CD34^+^ adult hematopoietic stem/progenitor cells, with native reticulocytes (nRET) and erythrocytes (nRBC) using cell biological and biomechanical technologies. To gain the desired all-encompassing information, a single method was unsatisfactory and only the combination of different methods could lead to the goal. Topographical information was matched with biomechanical data from optical tweezers (OT), atomic force microscopy (AFM) and digital holographic microscopy (DHM). Underlying structures were investigated in detail. Imaging, deformability and recovery time showed a high similarity between cRBC and nRBC. Young’s modulus and plasticity index also confirmed this similarity. No significant differences in membrane and cytoskeletal proteins were found, while lipid deficiency resulted in spherical, vesiculated cells with impaired biomechanical functionality. The combination of techniques has proven successful and experiments underscore a close relationship between lipid content, shape and biomechanical functionality of RBC.

## Introduction

Human erythropoiesis is a continuous differentiation process from hematopoietic stem/progenitor cells (HSPC) to enucleating orthochromatic erythroblasts that reside in the bone marrow. The enucleated, multi-lobular reticulocytes enter the circulation, where they eventually mature into biconcave erythrocytes (An and Mohandas, 2008; Griffiths et al., 2012; Mei et al., 2020). Some studies suggest an evolutionary advantage for reticulocytes to remain in the bone marrow until they are sufficiently mature and consequently adapted to the shear stress in the circulation system (Chasis and Schrier, 1989; Huisjes et al., 2018; Ovchynnikova et al., 2018). They distinguish three main events in terminal reticulocyte maturation, which include volume control, membrane remodeling and vesicularization (Huisjes et al., 2018; Ovchynnikova et al., 2018). Furthermore, the knowledge about terminal maturation has been enriched by thorough proteomics analyses (Gautier et al., 2016; Chu et al., 2018). Reticulocytes reduce their surface area (∼20%) and volume by expelling unneeded parts of the membrane and organelles through exocytosis, autophagy and re-arrangement of the cytoskeleton (Dzierzak and Philipsen, 2013; Minetti et al., 2018; Moura et al., 2018). In combination with the morphological changes, the deformability and membrane stability of the cells increase while becoming discocytes (Da Costa et al., 2001; Blanc et al., 2005). Based on this, maturing reticulocytes can be classified based on their morphology and shear moduli. The morphological stages can be depicted by Brillant Kresyl blue staining as well as by flow cytometry using anti-CD71/Thiazole Orange staining (Mel et al., 1977; Malleret et al., 2013; Ovchynnikova et al., 2018).

The so-called “R1” stage includes the nascent multi-lobular motile reticulocytes immediately after enucleation. The next stage (R2) contains less motile, deep-dish shaped granulated cells with a refractile ring and visible granules. The comparatively high stiffness and instability of immature reticulocytes could be firstly due to the larger surface area of multi-lobular cells, which presumably stretch the underlying spectrin filaments of the cytoskeleton. This increases the shear modulus of the membrane. Second, due to the staggered synthesis of the different cytoskeletal proteins, resulting in weak cohesion of the cytoskeleton and the superjacent lipid bilayer (Liu et al., 2010). A process of shortening of spectrin filament networks during reticulocyte maturation is described. Various studies state that the early reticulocyte stages are stiff, less stable than erythrocytes and easily fragmented under mechanical stress (Chasis et al., 1989; Chasis and Schrier, 1989; Ciana et al., 2017; Li et al., 2018). During further maturation in the circulation (R3) the granules disappear and the cells take on a more and more biconcave shape (R4) (Mel et al., 1977; Malleret et al., 2013).

Confirmed information on the exact timing of expression and synthesis of the major cytoskeleton proteins and their anchoring to the lipid bilayer during terminal maturation is still scarce. It is known that mutations and abnormalities in the two major complexes, the ankyrin and the junctional complex, lead to erythrocyte-specific diseases. Satchwell et al. found that the spectrin/ankyrin cytoskeleton plays a key role in hereditary spherocytosis arising from ankyrin deficiency, which negatively affects the expression of proteins like band 3, glycophorin A or spectrin (Satchwell et al., 2016). During reticulocyte maturation, cytoskeletal and plasma membrane proteins are massively rearranged. The cytoskeletal proteins required for subsequent erythrocyte function (spectrins, ankyrin, protein 4.1) and associated transmembrane proteins (band 3, glycophorin A) are preserved, some are reduced (cytosolic Actin), others are lost with the expulsed pyrenocyte (β1-integrin, erythroblast macrophage protein, adhesion molecules) (Mohandas and Evans, 1994; Van Den Akker et al., 2010; Lux, 2016; Ovchynnikova et al., 2018). Finally, erythrocytes possess a two-dimensional hexagonal lattice of spectrin tetramers linked by actin oligomers since a three-dimensional cytoskeleton would complicate the massive adaptations of RBCs in circulation (Jaferzadeh et al., 2018). Both the biconcave shape and deformability are essential for the survival and functionality of RBCs. Their biconcavity corresponds to the maximum surface area for the given volume and enables efficient gas exchange. The high deformability allows the RBC to pass through capillaries narrower than their own diameter in microcirculation (Buffet et al., 2006; Jaferzadeh et al., 2018). During ageing and in connection with pathologies such as sickle cell disease, sepsis or hereditary spherocytosis, RBCs lose their deformability and elasticity (An and Mohandas, 2008; Malleret et al., 2013; Moura et al., 2015; Huisjes et al., 2018). Consequently, these more rigid cells are retained in one of their multiple spleen passages, and phagocytosed by the red pulp macrophages, followed by degradation (Mebius and Kraal, 2005; Deplaine et al., 2011). This underscores the importance of the RBCs’ biomechanical properties for their persistence in the circulation.

Red blood cells are needed worldwide for safe and fresh blood products, but the increasing demand cannot be met exclusively by voluntary blood donation alone in the near future. The *ex vivo* generation of cRBC from different sources is on a promising path to clinical application (Douay, 2018). A first in man study was conducted in 2011 by Douay’s group (Giarratana et al., 2011). However, terminal remodeling to the biconcavity does still not occur completely *ex vivo*, raising the question of whether cRBC are as functional as nRBC in shape, structure, and deformability and amenable to clinical use. To answer this question, a deeper insight into similarities and discrepancies in the biomechanical behavior of native and cultured RBC (cRBC) is required. Understanding the underlying biomechanical properties could provide further insights to overcome the final hurdles to full functionality/maturation. New biomechanical studies mainly focus on mature nRBC, while comprehensive investigations on native reticulocytes (nRET) and cRBC are scarce. Furthermore, recent data showed that one technique is not able to cover the variety of cells in shape, functionality and structure (Bernecker et al., 2021). Each method has its limitations, and only a combination would allow the goal of an all-encompassing characterization to be achieved.

The aim of the present study is therefore to create an overall picture by comparing studies of nRBC, nRET and cRBC regarding the relationships between their shape and biomechanical properties related to the cytoskeletal and membrane protein expression using a portfolio of different technologies.

## Materials and methods

### Human specimen and ethics statement

Human erythrocytes were obtained from fresh RBC units within 24 h of donation. Human native reticulocytes were isolated from cord blood within 12 h post-partum with the CD71 Microbead kit according to manufacturer’s instructions (Miltenyi Biotec). CD34^+^ hematopoietic stem/progenitor cells (HSPCs) were purified from peripheral blood (purity 97.8 ± 0.7%) with the CD34 Microbead Ultrapure Kit (Miltenyi Biotec). Written informed consent was obtained from all volunteer donors prior to sampling. The study was approved by the local ethics committee of the Medical University of Graz, Austria, according to the Declaration of Helsinki (EK 27 165ex 14/15).

### Erythropoiesis culture and characterization

Erythroid differentiation from CD34^+^ HSPCs was performed according to our established three-phase culture model (Bernecker et al., 2019). Iscove’s basal medium (Biochrome) was supplemented with 5% human plasma from day 0 (Octapharma) (cRBC^w/o lipids^) or 5% human platelet lysate (cRBC^lipids^) (in house production UBT Graz) from day 8 onwards. All media were supplemented with 10 μg/ml insulin (SigmaAldrich) and 330 μg/ml human holo-transferrin (BBI solutions). Cells were induced to differentiation with 100 ng/ml stem cell factor (SCF), 5 ng/ml Interleukin-3 (IL-3) (both Peprotech) and 3 U/ml erythropoietin (EPO) (Erypo, Jansen Biologics B.V) and 10^–6^ M hydrocortisone (SigmaAldrich). The differentiation of the erythroid cells was monitored microscopically with May-Gruenwald-Giemsa (Hemafix, Biomed) and neutral Benzidine co-staining (o-dianisidine, SigmaAldrich). Additionally, the maturation stages were confirmed by flow cytometry (CD36, GPA, CD49d (Beckman Coulter); CD45, CD71 (Becton Dickinson); and band 3 (Bric 6, IBGRL, Bristol) on a CytoFLEX flow cytometer (Beckman Coulter). Dead cells were excluded by co-staining with 4.6-diamidino-2-phenylindole (DAPI; ThermoFisher). On day 18, cells were filtered through a syringe filter (Acrodisc, Pall) to obtain the pure enucleated RBC fraction free of precursors and expelled nuclei. The cRBCs were characterized again microscopically by Methylene Blue staining for ribosomal residues (Reticulocyte stain, SigmaAldrich) and by flow cytometry for CD71 expression and on the basis of Thiazole-orange stain (ReticCount, BectonDickinson).

### Scanning electron microscopy

For scanning electron microscopy, cells mounted on coverslips were fixed gradually with 0.1–1.0% glutaraldehyde in 0.1 M phosphate buffer pH 7.4 at room temperature for 1 h. Samples were post-fixed with 1% Osmiumtetroxid for 1 h at room temperature and then dehydrated in graded ethanol series (30–96 and 100% (vol/vol) EtOH). Critical point drying (Baltec CPD) and sputter coating (Baltec Sputter Coater 500) was applied. In addition, coverslips were placed on stubs covered with a conductive double-coated carbon tape. Images were acquired with a Sigma 500VP FE-SEM with a SEM Detector (Zeiss Oberkochen) operated at an acceleration voltage of 3 kV.

### Atomic force microscopy

Imaging- The RBCs were fixed in suspension with increasing concentrations of glutaraldehyde in PBS (0.1–0.5–1%) for 5 min at RT. Between the steps, the cells were centrifuged at increasing speeds (2200 U/min after the first fixation step, 4000 U/min in the second, 6000 U/min in the third step). After washing the RBCs in PBS and distilled water, 100 µL of this solution were pipetted on a 50 μg/ml Poly-L-Lysin (SigmaAldrich) coated Willco-dish (50/40). The cells were sedimented at RT for at least 30 min and then air-dried. The samples were imaged in tapping mode using the cantilever Tap300GD (BudgetSensors) with a resonate frequency of 300 kHz and a nominal spring constant of 40 N/m. Phaseolus vulgaris agglutinin–Erythroagglutinin (PHA-E, Sigma-Merck KGaA) was tested in concentrations of 0.1–100 μg/ml and incubation with 1 μg/ml for 30 min at RT identified as the best condition. Spectroscopy- Cultivated RBCs were washed twice with PBS by low-speed centrifugation steps (2200 U/min, 4 min, at RT) to remove for cultivation-important proteins that interfere with the attachment. Native RBC and cRBC were diluted to a concentration of 2 × 10^3^/ml in PBS and 100 µL were pipetted in the center of the Willco-dishes (50/40–WillcoWells) with cover glass bottom. The cells were allowed to sediment on the coated coverslips for 10 min in an incubator at 37°C, 5% CO_2_. After sedimentation, the media was changed to PBS/0.2% human albumin) and the cells were washed gently three times. The Willco dish filled with 3 ml of PBS/human albumin was placed onto the invert microscope in the 37°C warmed dish holder (CF prototype constructions at the Medical University of Graz) for observation and spectroscopy at the AFM.

Spectroscopy was performed in the static force mode using a Flex-Bio atomic force microscope (AFM) (Nanosurf) coupled to an optical microscope (Observer Z1, Carl Zeiss). Under physiological conditions, cells were observed in a warm liquid environment with a soft contact cantilever using a 2 µm spherical tip CP-qpSCont-PS-A (NanoAndMore GmbH) with a resonant frequency of 11 kHz and a spring constant of 0.01 N/m. The measurements were performed at a force of 300 pN and a velocity of 1 μm/s. For each experimental day, a new cantilever was calibrated before the experiment to obtain the spring constant using the software-integrated Sadder method. In liquid, the deflection sensitivity of each cantilever was measured on the cover glass as background and calculated in the AFM software. Data were collected with single force curves as horizontal and vertical lines or grids across the entire single cell. All lines and grids have been positioned to capture the background and the center of the cell. The force curves were calculated and evaluated with a formula using the analysis software SPIP (Image Metrology, Version 6.6.4), i.e.,

$$F_{Hertz}=\frac{4}{3}\frac{Ym}{\left(1-v^{2}\right)}\sqrt{R_{tip}{\left(s_{0}-s\right)}^{\frac{3}{2}}}$$

where Ym is the Young’s modulus, v is the Poisson’s ratio, Rtip is the ball radius of the tip in the Hertz model (Borin et al., 2017), s_0_ is the point of zero indentation, and s_0_-s denotes the indentation. Also, we have used the assumptions $Ym\ll Ym_{tip}$ , $s_{0}-s\ll R_{tip}$ , then no adhesion, and no viscoelasticity. To analyze the stiffness or cell elasticity of the cells, the Young’s modulus was calculated from the fitted curve with a Poisson’s ratio of 0.5. The use of this model is chosen when a rigid sphere intends a flat soft surface (Borin et al., 2017).

### Digital holographic microscopy

Cell morphology and cell membrane fluctuations (CMF) were measured for RBC in physiological solution, with the cells placed on a coverslip, using a custom DHM system based on a Mach-Zehnder interferometer (Bernecker et al., 2021). The morphological parameters: area, volume and sphericity were calculated from the cell height map as described (Bernecker et al., 2021). Area is defined as the projected area of the cell on the coverslip, volume is the volume of the cell, and sphericity is a coefficient related to the specific biconcave shape of the nRBC. The mean corpuscular hemoglobin (MCH) is calculated considering the projected area and the mean cell phase value. The wavelength of the laser light (LP520-SF15, Thorlabs Inc.) was 520 nm and the power at the sample was lower than 1 mW. The holograms were recorded with a sCMOS camera (CS2100M-USB, Thorlabs Inc.) and the reconstruction was performed by custom algorithms and code written in Matlab (MathWorks, Natick, MA). The refractive index of the cells was considered to be *n* = 1.418 (Jaferzadeh et al., 2018) and that of the physiological solution *n* = 1.33. The refractive index nRBC is considered the same for all the RBC types analyzed below. For CMF, a sequence of holograms was acquired for 3–4 s at 110 frames per second. The standard deviation (STD) of the cell height fluctuation was calculated for each pixel of the cell and background as described (Bernecker et al., 2021). The CMF value for a cell was calculated as the mean of the STDs for all cell pixels. The mean of the STD over the background pixels was calculate in the same way to evaluate the differences between the background and cell fluctuations.

### Optical tweezers

Cell deformation and cell membrane fluctuations of RBCs cells suspended in an optical trap in physiological solution at 37°C in a position far from the coverslip were studied using a custom optical tweezers setup as described in our previous works (Yousafzai et al., 2016; Falleroni et al., 2018; Bernecker et al., 2021). A single mode Yb fiber laser at 1,064 nm (YLM-5, IPG Photonics GmbH) (Yousafzai et al., 2016) was used for trapping and a Focused Tunable Lens FTL (EL-10–30-NIR-LD, Optotune AG) of which focal length can be precisely tuned was implemented to change the vertical position of the optical trap as described (Falleroni et al., 2018). The laser power at the sample plane was controlled between 0 and 80 mW. Cells suspended in physiological solution which are placed on the glass surface of the coverslip. A single cell was lifted by the laser beam and optically trapped at a height of about 20–25 µm from the surface. The FTL allowed us to decouple the imaging plane from the trapping plane. Cell trapping, deformation and shape recovery were monitored by time-lapse microscopy (×100 magnification, 500 fps) for 30 s and video recording using a high-speed camera (Fastec HiSpec 4, United States). The trapping time was measured from the time the laser was turned on until the cell was stably trapped. Stable trapping was defined by stable position and deformation of the cell. Recovery time was defined from the time the laser was turned off to the moment in which the cell shape recovery was observed. Cell deformation was observed during cell trapping for three different laser powers: 20 mW, 40 and 80 mW.

### Immunocytochemistry

For immunocytochemistry, 1.8 × 10^5^ cells per slide were sedimented on Poly-l-lysine coated (0.01%; SigmaAldrich) glass slides at 37°C. Samples were washed with buffer 1 (PBS with 1 mg/ml BSA (SigmaAldrich) and 2 mg/ml glucose (SigmaAldrich). One percent paraformaldehyde (ChemCruz) was used for fixation and 0.05% saponin (SigmaAldrich) for permeabilization. Antibody dilutions were made with buffer 1 plus 0.005% saponin, unspecific reactions were blocked with 10% goat serum (SigmaAldrich). Cells were incubated with anti-alpha 1 spectrin antibody, mouse monoclonal; anti-ankyrin erythroid/ANK antibody, mouse monoclonal; anti-band 3/AE 1 antibody, rabbit monoclonal; anti-non-muscle myosin IIβ/MYH10 antibody, rabbit monoclonal, or F-actin Staining Kit, respectively (all Abcam, 1:200). Secondary antibodies Alexa Fluor^®^ 405 Goat Anti-Rabbit (IgG) (ab175652) and Alexa Fluor^®^ 488 Goat Anti-Mouse (IgG) (ab150113) (both Abcam, 1:1,000) were diluted in 4% goat serum. Slides were covered with Vectashield Mounting medium (Prolong™ Glass Antifade Mountant, ThermoFisher Scientific) and coverslips for imaging on a Nikon eclipse Ti (60x with oil) with a Nikon A1 plus camera (pixel size 1,024, bit depth 8 bit). In more detail, the settings on Nikon eclipse Ti were for ankyrin HV 70, Laser 2.0, Zoom 2.392; for spectrin HV 80, Laser 3.0, Zoom 2.392, both green (488.9, emission 500–550); for band 3 HV 120, Laser 5.0, Zoom 2.392, blue (402.6, emission 425–475); for F-actin HV 120, Laser 4.0, Zoom 2.392, red (561.3, emission 570–620) and for myosin HV 130, Laser 5.0 and Zoom 2.392, blue (402.6, emission 425–475).

## Results

### Monitoring of erythroid differentiation

*Ex vivo* erythropoiesis was performed from CD34^+^ hematopoietic stem/progenitor cells under two different culture conditions, lipid supplementation or lipid starvation. Within the established 3-phase erythropoiesis culture (Giarratana et al., 2005; Bernecker et al., 2019), erythroid differentiation was monitored microscopically and by flow cytometry. On the last days of culture, >99% GPA + cells were detected in the homogenously differentiated cultures. After filtration, fractions with >99% purity of enucleated cRBC were collected for subsequent analyses. The corresponding maturity stages were analyzed using CD71 staining, ReticCount and New Methylene Blue staining. Overall, the maturation stage of cRBC was found to be in-between nRET and nRBC, and in agreement with recently published data from our group (Bernecker et al., 2019; Bernecker et al., 2021).

### Multimodal red blood cell imaging

**FIGURE 1:**
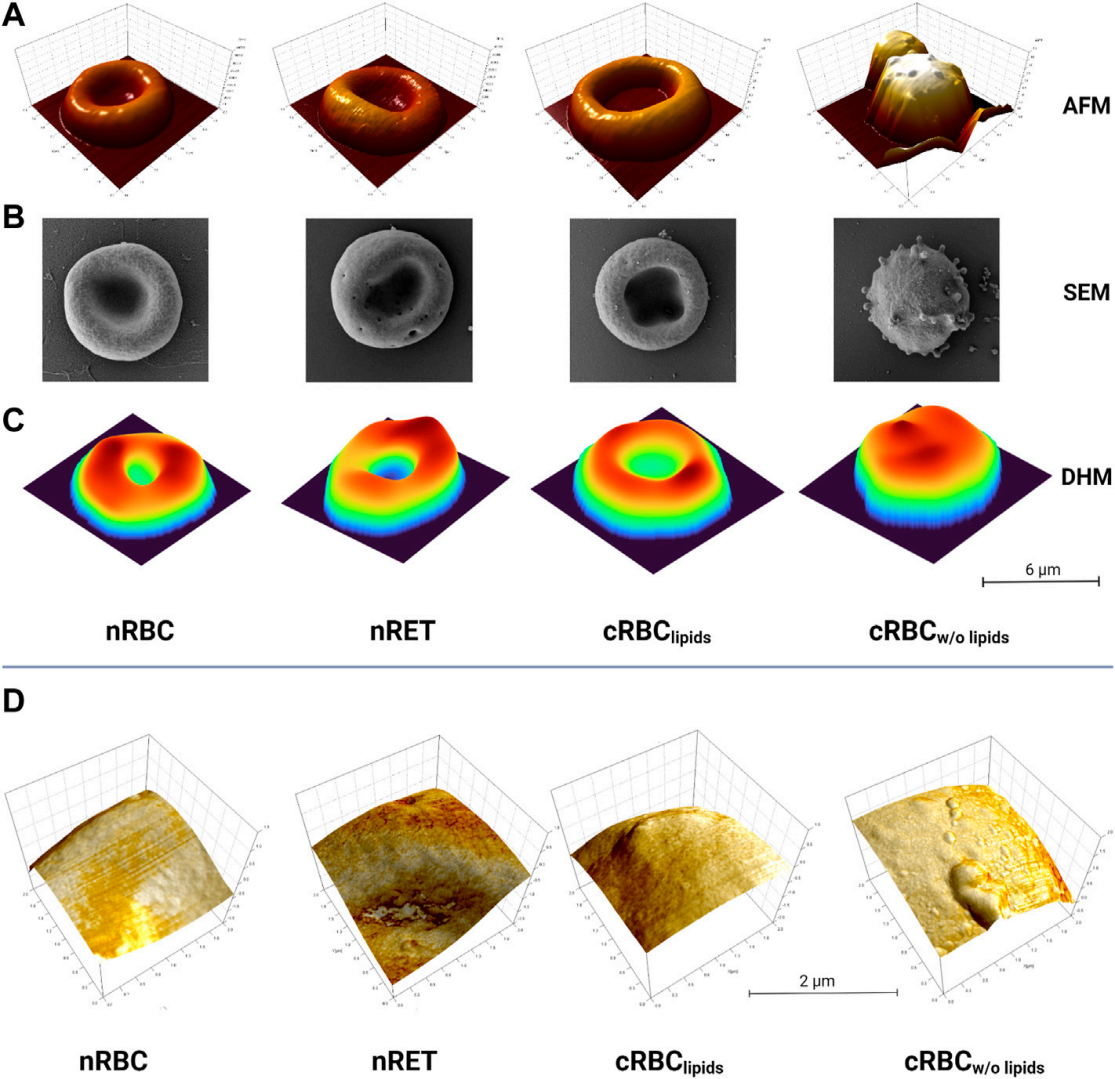
Imaging. Images of nRBC, nRET, cRBC^lipids^ and cRBC^w/o lipids^ using **(A)** AFM, **(B)** SEM and **(C)** DHM technology (scale bar is 6 µm). All imaging techniques reveal a nearly biconcave shape for cRBC^lipids^, widespread membrane vesiculation and a high grade of sphericity only for cRBC^w/o lipids^ compared to native red blood cells. **(D)** Detailed AFM images of cell surfaces of nRBC, nRET, cRBC^lipids^ and cRBC^w/o lipids^ depicting enhanced vesiculation of the membrane of lipid-poor cells, while the others do not show these peculiarities (scales: 2 μm × 2 µm). All images are representative images of *n* > 50 per cell type and imaging method.

**FIGURE 2:**
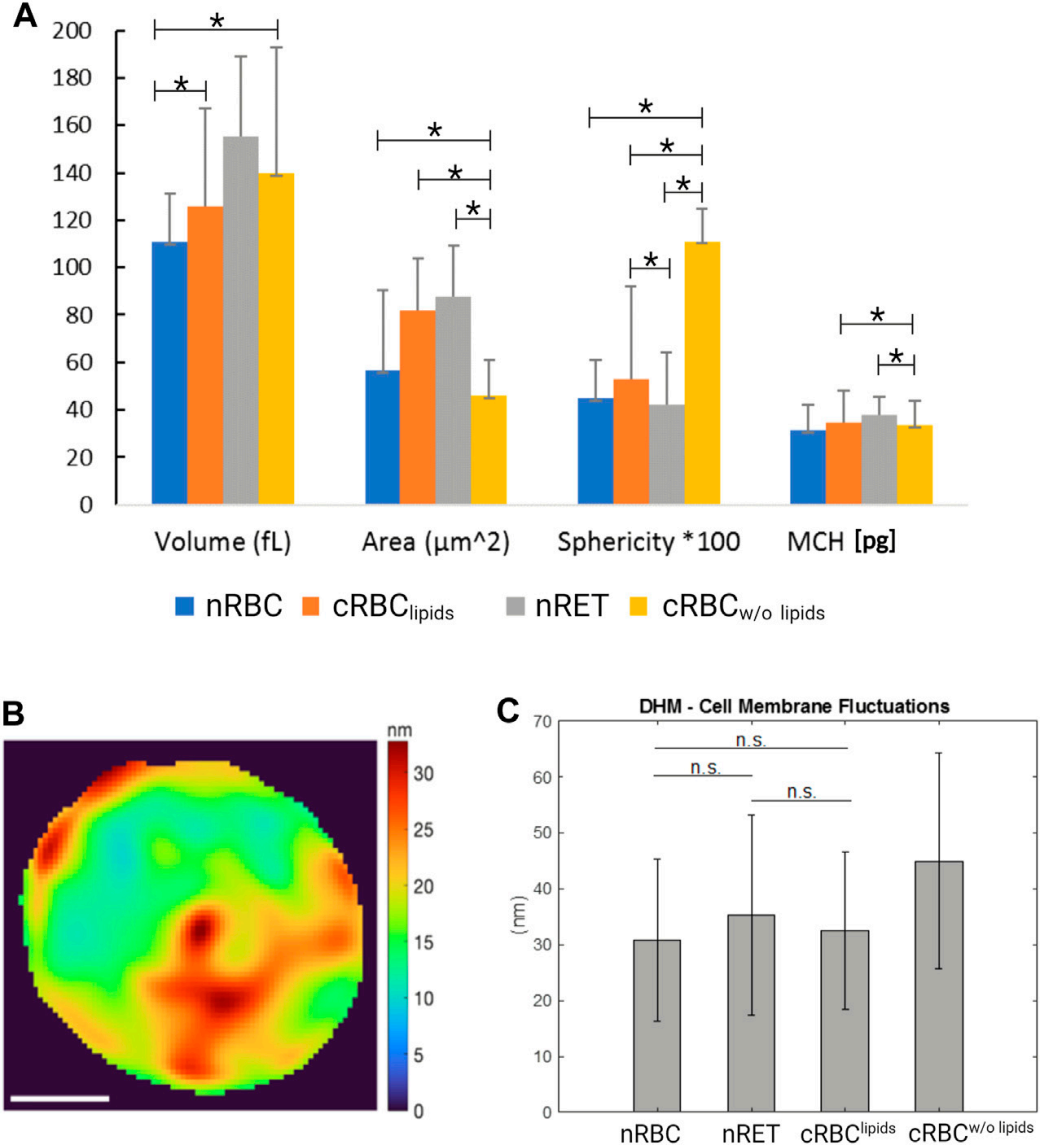
DHM analyses. **(A)** Cell morphological parameters (volume, area, sphericity) and MCH measured by DHM for: nRBC (*n* = 49), nRET (*n* = 46), cRBC^lipids^ (*n* = 52), cRBC^w/o lipids^ (*n* = 47). The units are given in femtoliter [fl] for volume, micron squared [μm^2^] for area and [g/dl] for MCH. The effective values for sphericity are multiplied by 100 for the representation in the graph. Mean ± standard deviation (*p < 0.05). **(B)** Cell membrane fluctuation (CMF) measured by DHM: example of CMF values over a cRBC^lipids^ cell (scale bar is 2 μm) **(C)** mean and std of CMF measured by DHM for nRBC, nRET, cRBC^lipids^ and cRBC^w/o lipids^.

AFM images (Figure 1A) show nRBC as the typical homogenous discocytes and the still immature nRET as more in-homogenous and multi-lobular due to their maturity stage. Throughout the experiments, cRBC^lipids^ showed a range of different maturity stages. Although not all cells become biconcave, images of a nearly biconcave example of cRBC^lipids^ is shown in Figure 1. In contrast, cRBC^w/o lipids^ are typically more spherical and highly vesiculated, indicating possible membrane defects. High resolution imaging of AFM revealed detailed images of the membrane surface of the cells, also referred to in Figure 1D. SEM imaging (Figure 1B) confirmed the AFM results, showing nRBC with homogenous, even surface, while nRET and cRBC^lipids^ display slightly more texture, like pits, blebs and vesicles indicating their more immature stage. In AFM and SEM, cRBC^w/o lipids^ show lots of vesiculation and blebbing, suggesting underlying membrane disorders rather than normal membrane remodeling during maturation. As shown in Figure 1, all three methods produce the same results. AFM and SEM reveal more details on the texture, since DHM provided the 3D information (Figure 1C) at a lower resolution than the other two techniques due the high resolution. On the other hand, DHM generated data on morphological parameters of the cells (Figure 2A) under the most natural conditions (physiological liquid environment, no adhesion to the substrate). The smallest cell projected area, calculated from DHM data, corresponds to the cRBC^w/o lipids^ (mean ± SD: 46 ± 14.8 µm^2^), which have also the biggest sphericity coefficient (1.11 ± 14) indicating a convex shape, nearly spherical. In fact, the volume (139.7 ± 53.1 fL) is large due to this shape. The largest projected cell area was found for nRET (87.5 ± 21.5 µm^2^), which also has the largest volume (155.4 ± 33.7 fL) and a sphericity (0.42 ± 0.2) indicating a dumbbell shape with a pronounced central concavity. The cRBC^lipids^ area (82.1 ± 21.5 µm^2^) is closer to the nRET than the nRBC area (56.7 ± 8.2 µm^2^). However, the cRBC^lipids^ volume (125.9 ± 41.3 fL) is closest to the nRBC (110.7 ± 20.4 fL). The sphericity (0.53 ± 0.4) for cRBC^lipids^ indicates a dumbbell shape similar to that of nRBC sphericity (0.45 ± 0.16), but with a slightly higher cell height at the central dimple. The mean corpuscular hemoglobin (MCH), calculated using the projected area, and the mean reconstructed phase are represented together with the morphological parameters in Figure 2. The mean MCH values are between 31.23 ± 11.2 pg for nRBC and 38.07 ± 7.32 pg for nRET. To compare the 4 groups, a non-parametric test for independent samples was performed (Kruskal–Wallis test with Bonferroni correction) for volume measurements, area, MCH, and sphericity. The most important differences were found for cRBC^w/o lipids^ compared to the other groups.

### Biomechanical functionality

**FIGURE 3:**
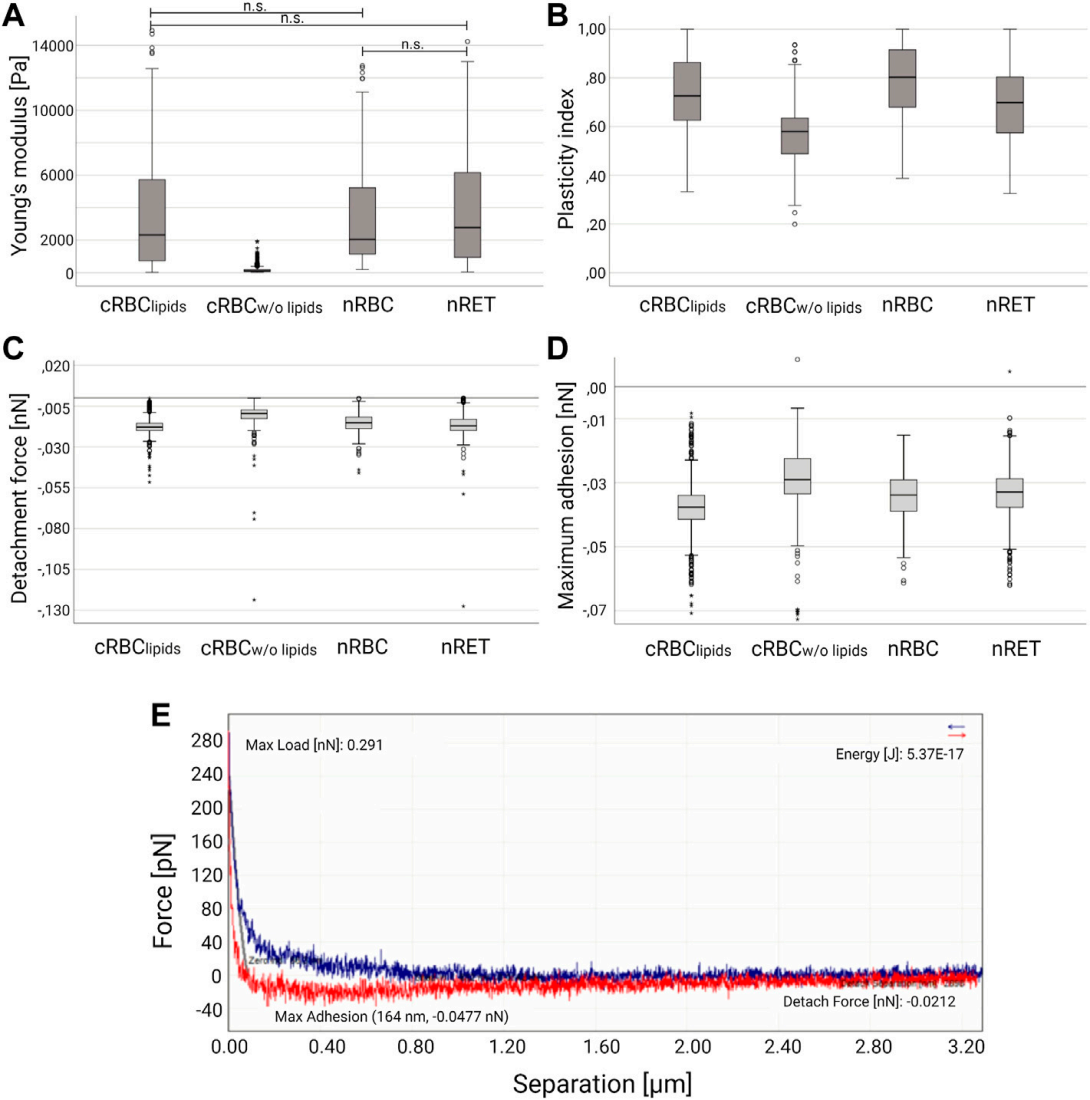
Atomic force microscopy spectroscopy. Box-whisker plots show differences in **(A)** Young’s modulus, **(B)** median plasticity index **(C)** detachment force and **(D)** maximum adhesion for nRBC (*n* = 625), nRET (*n* = 1,697), cRBC^lipids^ (*n* = 842) and cRBC^w/o lipids^ (*n* = 310). According to the statistical distribution of the data, a non-parametric test for independent samples was performed to compare the four groups (Kruskal–Wallis test with Bonferroni correction). The groups differ statistically significant (*p* < 0.05) unless otherwise stated (n.s.). **(E)** Representative force-separation curve (nRET).

In AFM spectroscopy, cRBC appeared more fragile than nRBC and nRET. With the exception of nRBC, all cell types showed variations in shape depending on the stage of maturation. The measurement of cRBC^w/o lipids^ was difficult as they did not adhere to the coating and often became detached from the cantilever. In addition, less mature cRBC also lacked the attachment to the glass surface, which made the measurements very difficult and required an increased number of experiments. The Young’s modulus (Ym) is a mechanical property that measures how easily the material can be bent or stretched. It is an important factor in determining the elasticity and deformation of a material, or its stiffness when an external force is applied, the greater the stiffer. It was calculated from the AFM force curves, using the Hertz model, where calculations yielded the highest median Ym for nRBCs (2.03 kPa). In connection with their different maturity grades, cRBC^lipids^ showed a similar behavior in AFM measurements with a median Ym of 2.32 kPa. Immature cRBC^lipids^ (lobular) had the lowest median Ym (1.50 kPa), while the median Ym for the most mature cRBC^lipids^ (biconcave) was 4.55 kPa. The same applied to nRET with a range between 2.85 kPa percentile 50 and 6.23 kPa percentile 75, depending on the maturity. The lipid starving cRBC^w/o lipids^ (spherical) showed a median Ym of 94.98 Pa and thus the lowest flexibility (Figure 3A).

**FIGURE 6:**
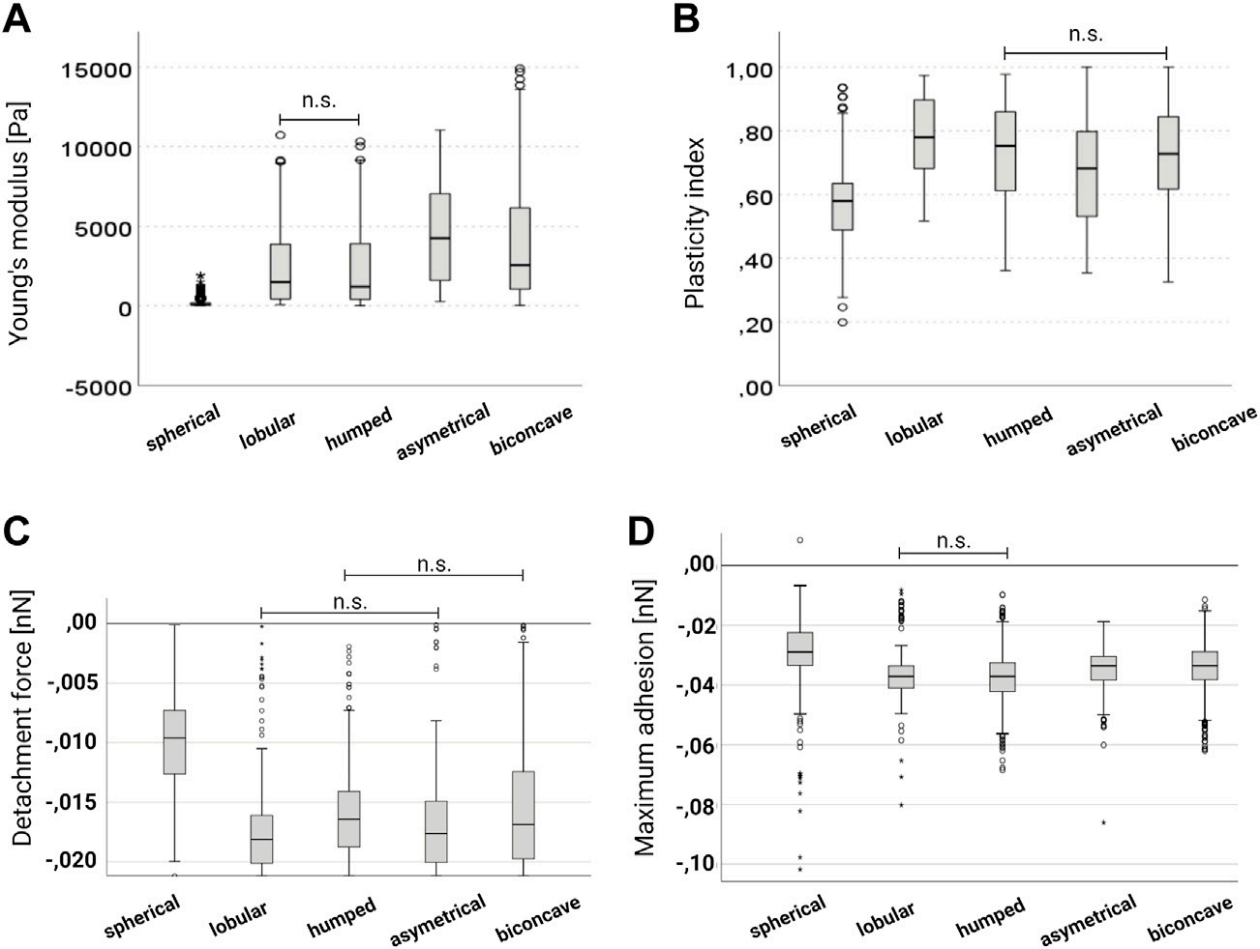
AFM analyses for different maturation stages. Box-whisker plots comparing **(A)** plasticity index, **(B)** Young’s moduli **(C)** detachment force and **(D)** maximum adhesion between the different maturation stages from immature, spherical (*n* = 310), lobular (*n* = 324), humped (*n* = 337), asymmetric (*n* = 550) to mature, biconcave (*n* = 1953). According to the statistical distribution of the data, a non-parametric test for independent samples was performed to compare the four groups (Kruskal–Wallis test with Bonferroni correction). The groups are statistically different (*p* < 0.05), unless otherwise stated (n.s.). Truncated whiskers and outliers in **(C)** are generated cropping the *Y*-axis for better visibility.

AFM deformation curves were further used to assess the short-time viscoelastic behavior of the cells, calculating the plasticity index [ζ = 1-(A2/A1)] for all groups. With ζ = 0 the cells are fully elastic, ζ = 1 means fully plastic behavior. Intermediate values 0 < ζ < 1 indicate viscoelastic behavior (33). Again, cRBC^lipids^ (median *ζ* = 0.726) are close to nRBC (median *ζ* = 0.803). In contrast to the Ym, plasticity indices (lobular 0.78, humped 0.71, asymmetric 0.65, and biconcave 0.69) were not as different between maturation stages within the same cell type (Figure 6). Native reticulocytes showed a median plasticity index of *ζ* = 0.699 (humped 0.797, asymmetric 0.687, symmetric 0.688). The lowest median plasticity index was found in cRBC^w/o lipids^ (spherical) with ζ = 0.580. Based on these results, nRBC and cRBC^lipids^ appear to be more plastic than nRET and cRBC^w/o lipids^, which might be due to their biconcavity especially when compared to the spherical shape of cRBC^w/o lipids^. In addition, analyses of detachment force and maximum adhesion revealed significant differences between all groups, the highest values for both were found in cRBC^w/o lipids^ suggesting higher membrane adhesiveness. Comparison of Young’s modulus, plasticity index, detachment force and maximum adhesion revealed by AFM spectroscopy of the 4 cell types are shown in Figure 3.

Cell membrane fluctuation using DHM was measured as described in the materials and methods. An example of cell fluctuation at different points in a cRBC cell is shown in Figure 2C. The cell fluctuation for a given point on the cell is expressed by the standard deviation of the membrane position (cell height), measured by DHM at a sampling frequency of f = 110 Hz. As can be seen from the figure, the cell fluctuation varies about 8–32 nm for different regions of the cell. The CMF is calculated as the mean of the cell fluctuation CMF over all points of the cell. The CMF values shown in Figure 2C indicate that nRBC (30.7 ± 20.4 nm) and cRBC^lipids^ (32.5 ± 14.1 nm) are very similar in terms of membrane flexibility. nRET have a larger CMF (35.3 ± 17.9 nm) but are still not significantly different from the other two. The largest CMF was found for the cRBC^w/o lipids^ (44.9 ± 19.2 nm).

### Cell deformation by optical tweezers

**FIGURE 4:**
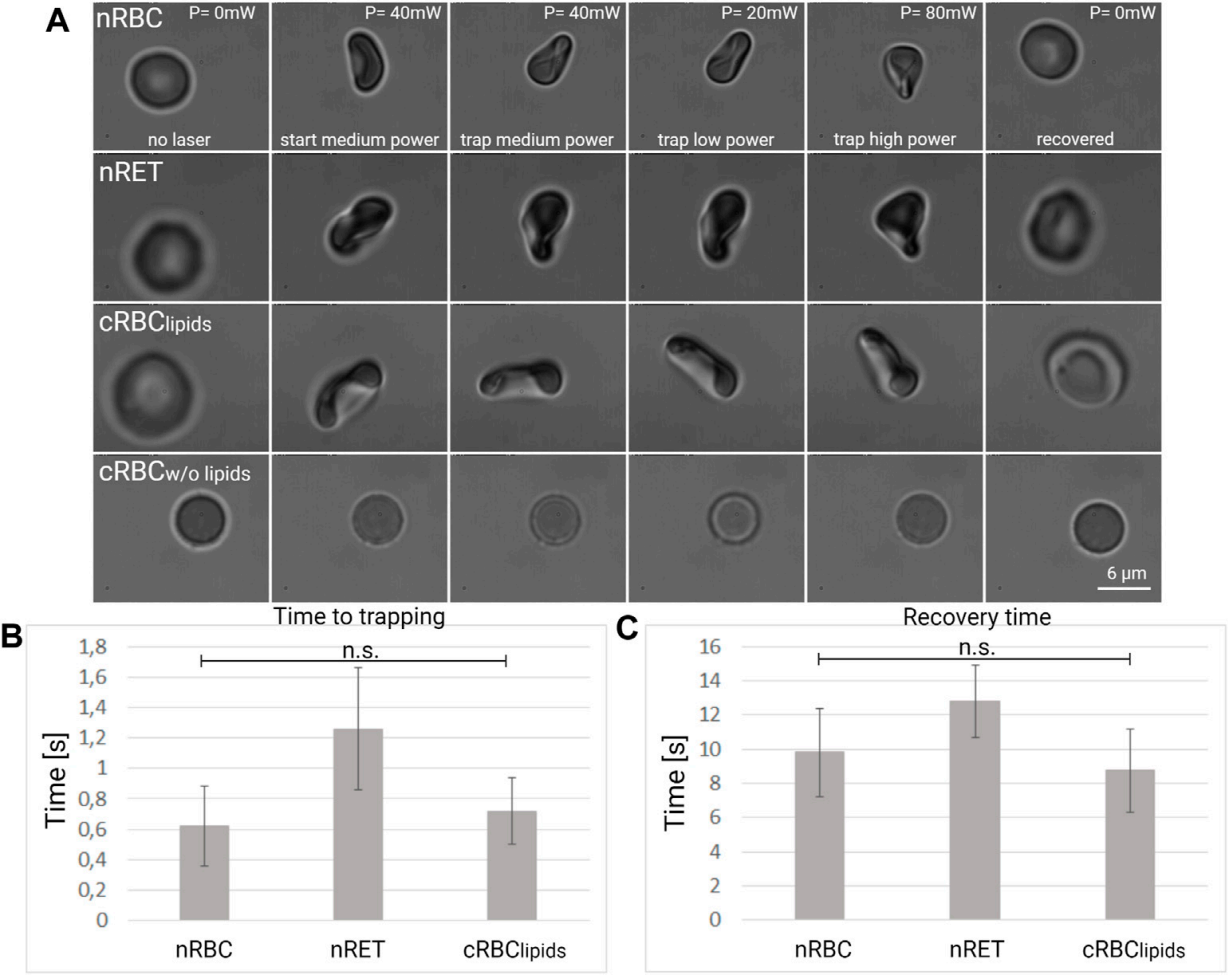
Cell deformation under optical forces. **(A)** Representative images of nRBC, nRET, and cRBC cell deformation under optical forces generated by the laser beam at low, medium and high power. The graph below shows a comparison of **(B)** time to trapping and **(C)** recovery time in seconds between nRBC (*n* = 27), nRET (*n* = 23) and cRBC^lipids^ (*n* = 25) in the optical trap. The graphs show the mean and the standard deviation.

Optical tweezers can exert pico-Newton forces on the cells, which can be deformed and trapped. We used the experimental protocol described in the methods section to investigate the deformation in single cell experiments. An example of the cell deformation under optical forces is illustrated for each cell type Figure 4A and in the (Supplementary Video S1). The cell placed first on the coverslip (laser off, *p* = 0 mW) was trapped by the laser tweezers (*p* = 40 mW) at a distance of about 20 μm from the coverslip surface. After 5 s of stable trapping, the optical forces were decreased, reducing the laser power (*p* = 20 mW) and the differences in the cell folding was observed. After another 5 s, the laser power was increased (*p* = 80 mW) to observe whether the cell retained its elasticity and was able to fold again. Finally, the laser was turned off and the cell was released from the trap and monitored if it regained its original shape. By observing the cells deformation at different optical forces, we found that different cells took on different shapes during folding: nRBC and nRET take on a typical bell shape, but nRET appears to fold less and with a more irregular shape. Cultured RBC^lipids^ appear to be more curved and nearly cylindrical in shape, while cRBC^w/o lipids^ maintain their spherical shape. Using a high-speed camera (500 fps), we measured the time to trapping and the recovery time, as described in the methods section. The results are shown in Figures 4B,C. The cRBC^w/o lipids^ were not considered for this analysis as they retained their spherical shape during optical manipulation. Native RBC and cRBC^lipids^ have similar values for trapping and recovery times (0.62 vs. 0.72 s for trapping, 9.8 vs. 8.75 s for recovery), suggesting similar deformation capabilities. The trapping and recovery times for nRET (1.26 s for trapping, 12.8 s for recovery) are higher and significantly different, suggesting that nRET are less resilient.

### Expression of membrane and cytoskeletal proteins during differentiation

**FIGURE 5:**
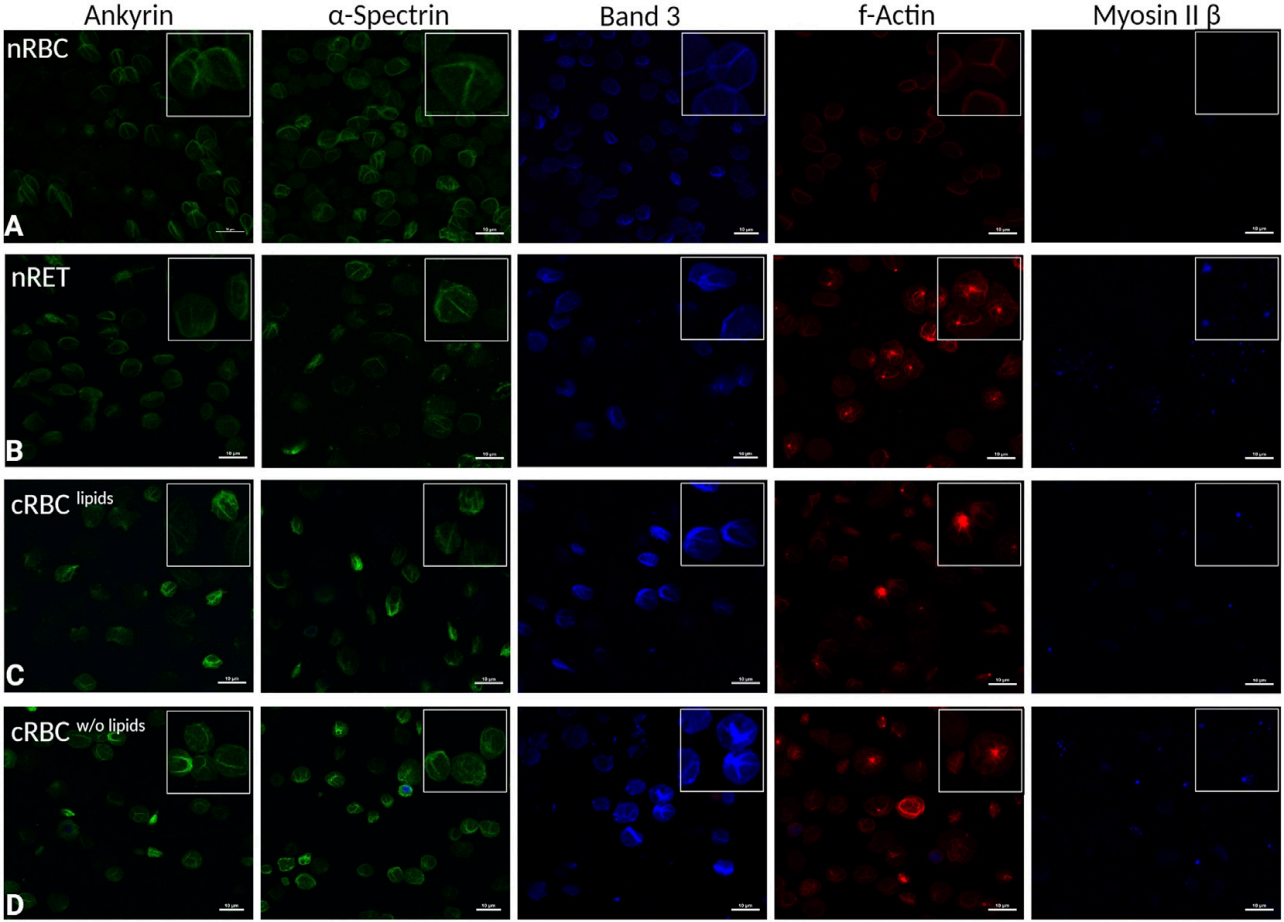
Immunocytochemistry. Representative fluorescence microscopy images of immunocytochemically stained cytoskeletal and membrane proteins from left to right: ankyrin, α-spectrin, band 3, F-actin, and myosin IIβ in **(A)** nRBC, **(B)** nRET **(C)** cRBC^lipids^ and **(D)** cRBC^w/o lipids^ (scale bars are 10 µm). In each of the images, there is a magnified section to show details of cells. All images are representative images of n > 25 per cell type.

To investigate possible effects on shape and biomechanical properties, the expression of the main membrane and cytoskeletal proteins ankyrin, α-spectrin, F-actin, myosin IIβ and band 3 was analyzed by immunocytochemical staining (Figure 5). Native RBCs (Figure 5A) showed typical segmented patterns in fluorescence microscopy. nRET (Figure 5B) and filtered day 18 cRBC^lipids^ (Figure 5C) revealed no clear differences in ICC staining of ankyrin, α-spectrin and only slight elevation of the membrane protein band 3. cRBC^w/o lipids^ (Figure 5D) displayed the most in-homogenous cell population also in the staining. Interestingly, F-actin showed accumulated spots in the immature cell types, nRET, cRBC^lipids^ and cRBC^w/o lipids^, which could be remnants of recent enucleation. In addition, myosin IIβ revealed clearly stained spots in immature cRBC^w/o lipids^ and nRET, few in cRBC^lipids^, while no blue staining was detected in mature nRBC.

### Maturation grade

Throughout the study, the maturation stage was an important issue for different cellular behavior in the different experiments. AFM and SEM analyses enabled at least slight fixation of cells, which enabled the analysis of immature cRBC^w/o lipids^ despite their membrane failure. For OT analyses, RBC with immature maturity grade and membrane speculative membrane defects, like mainly cRBC^w/o lipids^, were excluded by natural selection during pre-analytics. Therefore, for OT analyses mainly higher maturity and cells close to biconcavity were used. Figure 6 shows the cellular biomechanical properties according to their maturation grade revealed by AFM. Where the spherical group represents cRBC^w/o lipids^, while cRBC^lipids^, nRET and nRBC are among all the other stages of maturity. A comprehensive conclusion of all cellular characteristics is given in Table 1.

**TABLE 1:** Summary of the cellular characteristics. Volume, area, CMF, MCH, Young’s modulus, plasticity, trapping time and recovery of cRBC^lipids^ are located between values of nRBC and nRET. Diameter, height and sphericity are, however, more extreme than in nRET. The increased sphericity of cRBC might be linked to the increased band 3 and F-actin expression.

Cell type	nRBC	nRET	cRBC^lipids^	cRBC^w/o lipids^
Variety of shapes	biconcave	lobular, humped, asymmetrical, biconcave	lobular, humped, asymmetrical, biconcave	spherical, lobular, humped
**Diameter [µm] microscopy**	7.4 ± 0.32	8.2 ± 0.47	8,7 ± 0,35	9,4 ± 0,19
**Volume [µm** ^**3**^ **] DHM**	110.7 ± 20.4	155.4 ± 33.8	125.9 ± 41.3	139.7 ± 53.1
**Height [µm] DHM**	2.21 ± 0.5	1.99 ± 0.5	1.75 ± 0.4	3.33 ± 0.7
**Area [µm** ^**2**^ **] DHM**	56.7 ± 8.2	87.5 ± 23.5	82.1 ± 23.5	46.0 ± 14.8
**Sphericity DHM**	0.45 ± 0.2	0.42 ± 0.2	0.53 ± 0.4	1.11 ± 0.1
**CMF [nm] DHM**	30.7 ± 20.4	35.3 ± 17.9	32.5 ± 14.1	44.9 ± 19.2
**MCH [pg] DHM**	31.2 ± 11.2	38.1 ± 7.3	34.9 ± 13.4	33.5 ± 10.0
**Young’s modulus [kPa] AFM**	2.03	2.85	2.32	0.09
**Plasticity index AFM**	0.803	0.699	0.726	0.580
**Trapping time [s] OT**	0.61	1.25	0.75	n.a.
**Recovery time [s] OT**	10	12.5	8.5	n.a.

## Discussion

Blood transfusions are an irreplaceable life-saving therapeutic instrument. Since only erythrocytes from healthy voluntary blood donors are used, and their number is declining not only due to demographic changes, there is a growing supply bottleneck. Nonetheless, there is a risk of adverse reactions with blood mismatches, risk of iron overload in chronic transfusion-dependent patients, and risk of allo-immunization to name a few (Anstee et al., 2012). A source of homogenous, standardized young RBCs, such as the *ex vivo* generated RBC (cRBC), addresses at least some of these problems. *Ex vivo* erythropoiesis protocols are well established, but for future clinical use, cRBC have to meet high quality standards that demonstrate their similarity to native RBCs. A critical step in the *ex vivo* generation of RBCs remains the failure of the final differentiation of the enucleated reticulocyte into the biconcave erythrocyte. Complete maturation to a homogenous population of biconcave erythrocytes has not yet been achieved *ex vivo* (Minetti et al., 2018). This might be caused by the lack of splenic inter-endothelial slits or by other environmental conditions such as *ex vivo* shear stress. Studies proposed that remodeling reticulocytes are in the spleen for 1–2 days and undergo their final shedding steps to maturation (Li et al., 2021). Crosby et al. stated that the non-essential membrane parts get stuck as the reticulocyte passes through the inter-endothelial slits, amputate them and then are cleared by splenic macrophages, while the remodeled reticulocyte moves on (Crosby, 1982; De Back et al., 2014). From the beginning to the end of their lifespan, RBCs must squeeze countless times through extremely narrow slits and deform severely in order to survive. The importance of shear stress in circulation for terminal maturation has also been shown in *ex vivo* studies using tubing or transfusion into mice (Kupzig et al., 2017; Moura et al., 2018). Deformability and shape recovery are among the most important characteristics of RBC *in vivo*. This was also found by previous RBC studies, which identified the three most important RBC properties to withstand permanent mechanical burden without structural deterioration: 1) the geometry of RBC with the optimal surface-area-to-volume ratio, 2) the viscosity determined by intracellular hemoglobin content and hydration state and 3) the membrane deformability/composition (Mohandas and Evans, 1994; Mohandas and Gallagher, 2008).

In the current study, we focused on these properties and compared *ex vivo* generated cRBC to native erythrocytes (nRBC) and reticulocytes (nRET) using cutting edge technologies for biomechanical characterization matched with data on shape and cytoskeletal protein expression.

Accurate measurement of cell shape and volume as well as hemoglobin concentration has been addressed in our recent studies (Bernecker et al., 2019; Bernecker et al., 2021) where mean corpuscular/cellular hemoglobin (MCH) revealed a high similarity between cRBC^lipids^ and nRBC, while the mean volume of cultured cells was higher than that of nRBC. Because MCH is one of the key factors in cytoplasmic viscosity, high values are known to decrease RBC deformability. However, the values measured in the 4 cell types should not have any increasing influence on the viscosity, as they do not significantly differ. Again, the larger diameter and volume of cRBC compared to nRBC might in part be explained by the lack of a spleen *ex vivo*. Clinical studies revealed that the nRBC of splenectomised patients were larger than those of healthy ones, while the size of their immature reticulocytes did not differ (Li et al., 2021). In particular, the abnormal, lipid starving cRBC^w/o lipids^ showed a significantly larger diameter, height and thus sphericity than the other cells, which was also confirmed by the imaging techniques SEM, DHM and AFM. Accordingly, Kuchel et al. found that the typical biconcave shape of healthy RBC allows volume and surface area preservation during deformation in very small capillaries (Kuchel et al., 2021). A spherical shape would be less easy to retain. Nonetheless, ageing RBCs become more spherical by losing surface area, resulting in a reduction of the surface-area-to-volume ratio. At a critical degree of sphericity, deformability is lost and RBC are eliminated from circulation. Diseases such as hereditary hemolytic anemia are characterized by altered nRBC deformability and reduced O_2_ transport (Huisjes et al., 2018).

In addition, possible regulators such as dehydration, cytoskeletal integrity, metabolism and membrane protein phosphorylation are discussed (De Back et al., 2014). Xu and others further showed that ATP metabolism affects membrane stiffness in stored RBCs. While the lack of ATP increases the spectrin-membrane affinity, leading to increased RBC stiffness, ATP re-synthetization induces dynamic spectrin-membrane binding and makes the RBC more deformable (Park et al., 2010; Xu et al., 2019). Sophisticated methods for quantitatively estimating the elastic modulus of the membrane-skeleton complex have gained scientific interest (Yeow et al., 2017). One upcoming method is AFM. Various studies on RBC in health and disease have been published, but the data differ as many groups used cell fixation procedures that massively affect cellular membrane behavior. Because the force-separation curve describes, strength, stiffness and elasticity of the cell surface (Yeow et al., 2017), available results from previous studies on nRBC might be compromised by fixation and must be viewed with caution. Although analysis of suspension cells is challenging, we established a protocol for RBC spectroscopy analyses under physiological conditions. In addition to the implementation of a fixation-free protocol, to the best of our knowledge this is one of the few studies looking at nRET and cRBC in comparison to nRBC in AFM (Chu et al., 2018). As expected, the degree of maturity influences the plasticity index and the Young’s modulus. Findings from imaging and morphological studies on the similarity of cRBC^lipids^ and nRBC have also been strengthened in AFM spectroscopy. The detachment force, the force measured for the last point of contact between the tip and the surface on the retraction curve, was highest for cRBC^w/o lipids^. The same was found for the maximum adhesion, defined by the closest point of contact and the relative force between the tip and the surface on the retraction curve. This might indicate that cRBC^w/o lipids^ are the stickiest. The observation is supported by publications by Malleret and others, who stated that stickiness of reticulocytes decreases with maturation (Chamberlain and Lichtman, 1978; Malleret et al., 2013). Here, too, not only the degree of maturity but also the lipid content of the membrane seems to play an important role, since cRBC^w/o lipids^ behaved significantly different from all other cells and exhibited extremely low Young’s moduli. The lowest plasticity index of the lipid-poor cells seemed contradictory at first, but might be explained by a sticky behavior during the analyses (Li et al., 2018). Interestingly, a recent AFM study by Li et al. documented that reduced connectivity or extension of the cytoskeleton might be the underlying reason for the increased shear modulus and higher stiffness of immature reticulocytes (Li et al., 2018). Presumably, due to lower resolution, we have not been able to reproduce these findings on the connectivity of lipid bilayer and cytoskeleton so far. Kozlova et al. also found that the Young’s moduli of stored RBC were higher than fresh cells, indicating higher stiffness, which is partially consistent with our results for spherical lipid-poor cRBC (Kozlova et al., 2017). The AFM results were confirmed by supplementary optical tweezer analyses with forces in the pico-Newton range, in which the properties, deformability, folding and recovery times of the cells in interaction with the radiation pressure of the light were examined. OT studies on red blood cells are emerging, but again, data on nRET and cRBC are still scarce. In the current study, Young’s modulus values for nRET, cRBC^lipids^ and nRBC measured by AFM were found to be similar, with slightly higher values for nRET, although not significant. However, as the OT experiments show, nRET behave differently than erythrocytes. This might be due to their multi-lobular shape and, although not confirmed here, a highly stretched and unfinished cytoskeletal structure. The time to trapping and recovery time for cRBC^lipids^ and nRBC were similar and significantly shorter than nRET, suggesting that nRET is less resilient. cRBC^lipids^ showed a folding shape similar to nRBC, as well as both deformability and recovery times when being trapped. Only cRBC^w/o lipids^ did not fold under pico-Newton optical forces due to their spherical shape. Cell membrane fluctuation analyses showed high CMF similarities between nRBC, nRET and cRBC^lipids^, while CMF is different for cRBC^w/o lipids^. However, this value is affected by the spherical shape of the cell and its height, as discussed in our recent publication (Bernecker et al., 2021).

Jaferzadeh et al. used digital holographic microscopy (DHM) to analyze changes in the fluctuation rates of RBC due to storage and found an increasing stiffness with ageing and storage (Jaferzadeh et al., 2018). Asymmetric lipid distribution within the plasma membrane plays a prominent role in RBC functionality. The membrane properties can be changed by the modification, the ion and water transport can be impaired, which in turn affects the density distribution (Kuypers, 2008). It is further known that cohesion between lipid bilayer and skeletal proteins prevents membrane vesiculation, while intracellular calcium increase and RBC aging stimulates membrane vesiculation and shedding of specific lipids and proteins (Nicolas et al., 2003; Willekens et al., 2008). *In vivo*, extended vesiculation leads to decreased deformability and eventually to trapping and premature elimination of RBCs in the spleen. In a recent study, we have already shown the effect of cellular cholesterol content on the biomechanical properties of RBC such as elongation and osmotic resistance (Bernecker et al., 2019). Interestingly, despite notable differences in the cellular biomechanical behavior throughout the study, no significant differences in cytoskeletal protein expression could be detected between the groups using immunocytochemical staining. No extended cytoskeleton or differences in spectrin and ankyrin expression were found, which might be due to final formation of the cytoskeleton in all cell types. Only in some of the slides, slightly higher band 3 staining was found in more immature cells. Remarkably, the more immature cell types, nRET, and cRBC, showed marked accumulation of F-actin and myosin IIβ spots that mature nRBC lacked. According to Mei et al., who found F-actin and non-muscle myosin IIβ along with non-muscle myosin IIα and other factors form the central network for cytoskeleton modulation during enucleation, these might be residual structures of recent enucleation events (Koury et al., 1989; Barr and Gruneberg, 2007; Moura et al., 2018; Mei et al., 2020). Thereafter, their function is no longer required and F-actin and myosin IIβ are removed during terminal erythroid maturation (Mohandas and Evans, 1994; Liu et al., 2010; Liu et al., 2011). Western blot analyses of the membrane and cytoskeleton proteins revealed no differences in expression levels between nRBC, nRET and cRBC (data not shown). This is consistent with Li et al. who found the same protein expression levels in nRET and nRBC in relation to spectrin and ankyrin (Bell et al., 2013; Chu et al., 2018; Li et al., 2018).

Deep investigations of RBC properties are extremely important to further elucidate the missing steps in shape remodeling. A better understanding of the biochemical and biophysical properties could improve the *ex vivo* culture process and thus the quality and storability of cRBC for transfusion purposes. In this study, we examined shape, biomechanical behavior, and cytoskeletal protein expression of cRBC compared to native reticulocytes and erythrocytes. Taken all discussed observations together, the results confirm a near perfect terminal maturation of cRBC comparable to nRBC in terms of cell morphology and biomechanical characteristics. Although this comprehensive study points a good path towards future clinical application, deeper investigations of the molecular interaction of lipid composition and membrane proteins, as well as investigations on the cellular ATP metabolism are necessary. Besides, the established methods may gain additional importance for diagnosing RBC pathophysiology in a large number of diseases.

## Data Availability

The raw data supporting the conclusions of this article will be made available by the authors, without undue reservation.
