# Supplementary material for: Biomechanical properties of native and cultured red blood cells–Interplay of shape, structure and biomechanics
Source: Front Physiol. 2022 Aug 16;13:979298. doi: 10.3389/fphys.2022.979298 (PMC9424772; doi:10.3389/fphys.2022.979298)
Supplement: Supplementary file 1 [file DataSheet1.PDF]

Supplementary Material

1 Supplementary Video

Supplementary Video 1. Representative video of a native red blood cell (nRBC). Cell trapping, deformation and shape recovery were monitored by time-lapse microscopy (100X magnification, 500 fps) for 30 s and video recording using a high-speed camera (Fastec HiSpec 4, USA). Cell deformation is shown during cell trapping for laser powers: 20 mW, 40 mW and 80 mW.
